# Supplementary material for: Causal Relationship between Aging and Anorexia Nervosa: A White-Matter-Microstructure-Mediated Mendelian Randomization Analysis
Source: Biomedicines. 2024 Aug 16;12(8):1874. doi: 10.3390/biomedicines12081874 (PMC11351342; doi:10.3390/biomedicines12081874)
Supplement: Supplementary file 1 [file biomedicines-12-01874-s001.zip › Supplementary Figure/Figure S4/Singe SNP plot of MR analysis for TL to ubm-b-1716.pdf]

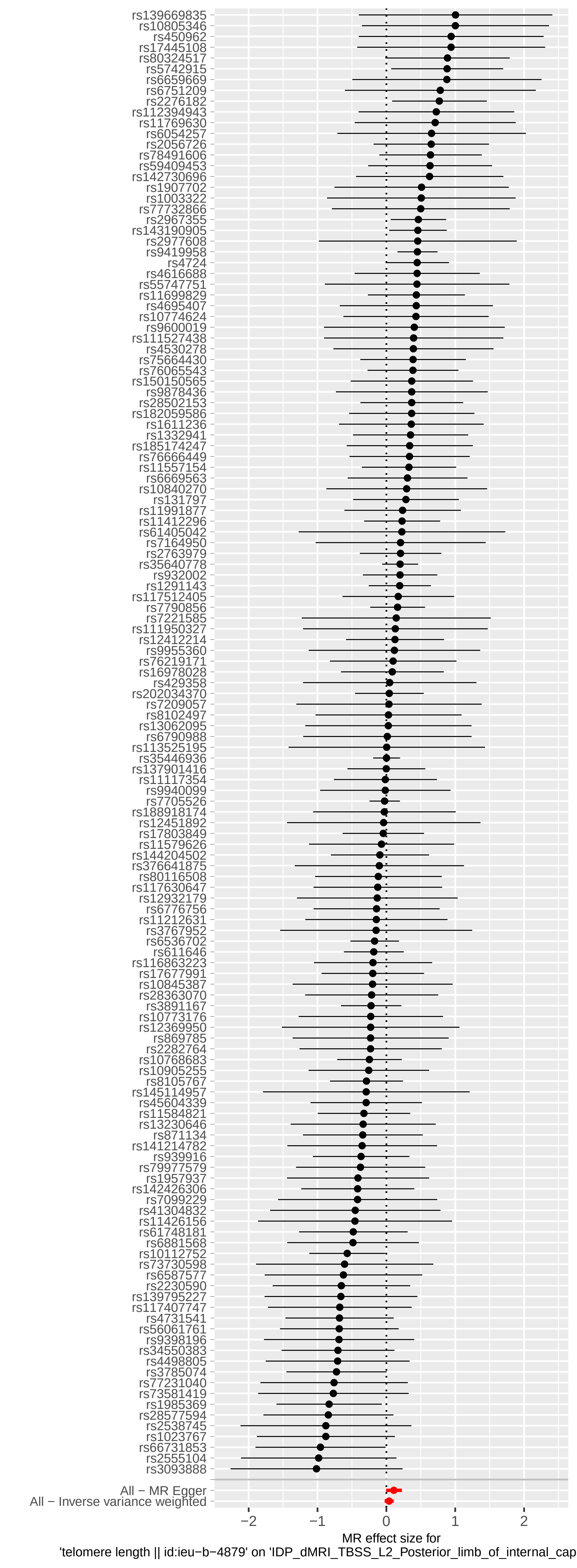
rs139669835
rs10805346
rs450962
rs17445108
rs80324517
rs5742915
rs6659669
rs6751209
rs2276182
rs112394943
rs11769630
rs6054257
rs2056726
rs78491606
rs59409453
rs142730696
rs1907702
rs1003322
rs77732866
rs2967355
rs143190905
rs2977608
rs9419958
rs4724
rs4616688
rs55747751
rs11699829
rs4695407
rs10774624
rs9600019
rs111527438
rs4530278
rs75664430
rs76065543
rs150150565
rs9878436
rs28502153
rs182059586
rs1611236
rs1332941
rs185174247
rs76666449
rs11557154
rs6669563
rs10840270
rs131797
rs11991877
rs11412296
rs61405042
rs7164950
rs2763979
rs35640778
rs932002
rs1291143
rs117512405
rs7790856
rs7221585
rs111950327
rs12412214
rs9955360
rs76219171
rs16978028
rs429358
rs202034370
rs7209057
rs8102497
rs13062095
rs6790988
rs113525195
rs35446936
rs137901416
rs11117354
rs9940099
rs7705526
rs188918174
rs12451892
rs17803849
rs11579626
rs144204502
rs376641875
rs80116508
rs117630647
rs12932179
rs6776756
rs11212631
rs3767952
rs6536702
rs611646
rs116863223
rs17677991
rs10845387
rs28363070
rs3891167
rs10773176
rs12369950
rs869785
rs2282764
rs10768683
rs10905255
rs8105767
rs145114957
rs45604339
rs11584821
rs13230646
rs871134
rs141214782
rs939916
rs79977579
rs1957937
rs142426306
rs7099229
rs41304832
rs11426156
rs61748181
rs6881568
rs10112752
rs73730598
rs6587577
rs2230590
rs139795227
rs117407747
rs4731541
rs56061761
rs9398196
rs34550383
rs4498805
rs3785074
rs77231040
rs73581419
rs1985369
rs28577594
rs2538745
rs1023767
rs66731853
rs2555104
rs3093888
All − MR Egger
All − Inverse variance weighted
−2
−1
0
1
2
MR effect size for
'telomere length || id:ieu-b-4879' on 'IDP_dMRI_TBSS_L2_Posterior_limb_of_internal_cap
